# Effect of Synthesis Routes and Support Nature on Co-Based Catalysts for Low-Temperature Catalytic Combustion of Methane

**DOI:** 10.1021/acsomega.5c10242

**Published:** 2026-04-06

**Authors:** Mirza Belal Beg, Labeeb Ali, Suryamol Nambyaruveettil, Abbas Khaleel, Mohammednoor Altarawneh

**Affiliations:** † 11239 United Arab Emirates University, Department of Chemical and Petroleum Engineering, Sheikh Khalifa bin Zayed Street, Al- Ain 15551, United Arab Emirates; ‡ United Arab Emirates University, Department of Chemistry, Sheikh Khalifa bin Zayed Street, Al- Ain 15551, United Arab Emirates

## Abstract

Catalytic combustion of methane (CH_4_) is one of the promising methods to tackle the emission of CH_4_ through natural gas-based vehicles. Herein, to study the impact of different preparation methods and support, a series of monometallic cobalt-based catalysts was prepared to examine the total oxidation of CH_4_ at low temperatures. With a 20% total metallic loading, the samples were synthesized using two different methods: wet impregnation and coprecipitation, with two different types of support, such as cerium oxide (CeO_2_) and silica oxide (SiO_2_). All prepared samples were characterized using several techniques to examine their physicochemical properties, such as XRD, Raman, FTIR, SEM-EDS, XPS, H_2_-TPR, and O_2_-TPD, which confirmed the crystalline phases and structural integrity of the catalysts. Structural and surface analyses confirmed that Co incorporation into CeO_2_ generated oxygen vacancies and stabilized Co^3+^ with high oxygen mobility and reducibility, whereas SiO_2_-supported catalysts showed weaker dispersion and limited redox activity. The catalytic performance of all prepared samples was investigated in the temperature range of 250–600 °C, and CeO_2_-based catalysts synthesized through the wet impregnation method exhibited 91% conversion at 600 °C, which is 25% higher than that of their SiO_2_-based counterpart due to the formation of oxygen vacancies, which enhanced the catalytic activity. The robustness of the best-performing catalyst was tested by varying the WHSVs to check the performance under real conditions. The findings from this study pave the way for the development of low-temperature catalytic processes for methane oxidation.

## Graphical abstract

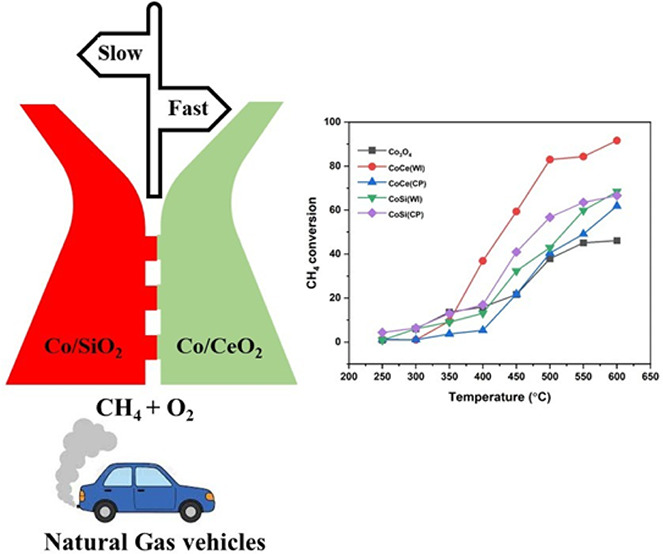

## Introduction

The main component of natural gas is methane (CH_4_), which is extensively used as a fuel in power plants and natural gas-based vehicles. However, CH_4_ is considered a potent greenhouse gas with a global warming potential approximately 22 times greater than that of carbon dioxide (CO_2_), and its emissions from natural gas systems, agriculture, and other sources cause a serious impact on human and environmental health.^1^ Consequently, it is become necessary to abate CH_4_ for sustainable development and environmental protection. The conversion of CH_4_ into less toxic compounds such as CO_2_ and H_2_O through combustion or oxidation reaction has emerged as a promising strategy for the mitigation of CH_4._ However, conventional thermal combustion of low-concentration CH_4_ typically requires temperatures above 1000 °C, mainly due to the fact that the CH_4_ molecule consists of very stable tetrahedral C–H bonds that are difficult to break,^2,3^ and such high temperatures often lead to the formation of undesired byproducts such as NO_*x*_ and CO.^4,5^ In contrast, catalytic combustion can achieve nearly complete oxidation of CH_4_ by utilizing a suitable catalyst at significantly lower temperatures.^6–8^

Generally, noble metal-based catalysts such as Pd and Pt are known to activate CH_4_ at relatively low temperatures, but their high cost and susceptibility to poisoning remain serious drawbacks.^9,10^ As the high cost of precious metals, there is significant interest in developing alternative, cost-effective catalysts based on different catalytic systems. In this regards, non-noble metals oxides such as CeO_2_,^7,11^ SiO_2_,^12^ and Al_2_O_3_ ^13^ have attracted considerable interest for the complete oxidation of CH_4_, not only because of their low cost but also due to their unique electronic structure in which their empty 3d orbitals allow them to interact with ligands, accept electrons from reactant molecules, and facilitate charge transfer processes, thereby promoting high dispersion and catalytic efficiency.^14^ Among transition metal oxides (TMOs), cobalt oxide (Co_3_O_4_) with a typical spinel structure has emerged as a promising alternative that is widely used for the combustion of CH_4_, due to its high catalytic activity resulting from its crystal structure and electronic properties.^1^ Typically, in Co_3_O_4_, cobalt shows variance in its oxidation states, with Co^2+^ in tetrahedral sites and Co^3+^ in octahedral sites, which facilitates redox reactions.^15^ Additionally, the presence of surface lattice oxygen and the relatively low Co–O bond energy contribute to its high turnover frequency during methane oxidation, which makes Co-based catalysts promising for oxidation reactions.^16^ In the literature, it is well mentioned that the incorporation of Co_3_O_4_ as active sites onto supported metal oxides, which have high surface areas, results in improved catalytic performance and long-term stability for CH_4_ oxidation.^17^ For instance, Co_3_O_4_ supported on different metal oxide support such In_2_O_3_,^18^ Al_2_O_3_,^19^ SnO_2_ ^20^ and ZrO_2_,^21^ efficiently showed improved catalytic activity and thermal stability for CH_4_ oxidation due to the interaction with doped metals which weakens the Co–O bond, thereby facilitating more oxygen mobility and promotes the formation of active oxygen species, thereby enhancing the catalytic performance.^22^ However, the strong interaction between the metal oxide and the support sometimes leads to inactive phases like cobalt aluminate and cobalt–magnesium oxides, which affects the catalytic activity.^23^ Thus, choosing an appropriate support is essential to maximize activity and prevent the formation of inactive phases in oxidation reactions.

It is known that the overall effectiveness of TMOs-based catalysts for CH_4_ oxidation is not only influenced by the choice of active phase. Moreover, it is strongly influenced by several factors, including the nature and intrinsic properties of the support, the metal loading, the size, morphology, as well as the preparation methods that determine the final catalytic activity and stability.^24,25^ As mentioned, the nature of the supported oxide plays an important role in determining the performance of the catalyst. In a recent study, Feng et al.^26^ prepared a series of catalysts by incorporating different weight loadings of Co_3_O_4_ on SmMn_2_O_5_ (SMO) support, where the 50% Co/SMO sample exhibited better activity and stability under oxygen-rich conditions. In another study, Co_3_O_4_ supported on different supports, such as γ-Al_2_O_3_, which was prepared through two preparation methods, such as wet impregnation and combustion methods, demonstrated complete CH_4_ oxidation at 350 °C due to their high Co^3+^ value and dispersion.^23^ In addition, Co_3_O_4_ supported on SnO_2_ oxides with a different Co/(Co+Sn) molar ratio, in which 0.75 exhibited the highest activity due to strong Co–Sn interactions that enhanced oxygen mobility, thereby improving catalytic performance.^20^ The other support oxide, such as cerium oxide (CeO_2_), used in Co_3_O_4_-based catalysts, exhibited better performance due to its redox ability to switch between Ce^4+^ and Ce^3+^, which facilitates the generation of oxygen vacancies and high oxygen storage capacity.^27^ This property significantly enhances the reducibility of the active phase, thereby promoting oxygen mobility and catalytic activity during CH_4_ oxidation. In literature, researchers have widely used CoO_*x*_/CeO_2_ catalysts for the complete oxidation of CH_4_, showing promising catalytic activity due to the strong redox interaction between cobalt and CeO_2_. For instance, Liotta et al.^28^ and Li et al.^30^ reported that CoO_*x*_ species supported on CeO_2_ exhibited low-temperature activity, which was mainly attributed to the facile Ce^4+^/Ce^3+^ redox cycle that enhances oxygen mobility and surface reactivity. Later, Li et al.^30^ prepared a cobalt-supported ceria catalyst by using a modified sol–gel method that demonstrated the synergistic Co–Ce interface promotes the formation of active oxygen species responsible for the complete oxidation of CH_4_. Similarly, Dou et al.^6^ emphasized the importance of interfacial lattice oxygen between Co_3_O_4_ and CeO_2_, which promotes CH_4_ conversion at a lower temperature. These studies establish CoO_*x*_/CeO_2_ as one of the most effective non-noble catalytic systems for CH_4_ combustion.

In contrast, SiO_2_ is generally considered an inert support, but it offers high surface area, uniform pore distribution, and excellent thermal stability, which can favor high dispersion of active species.^31^ However, due to its limited redox capacity, SiO_2_-supported catalysts depend mainly on surface-adsorbed oxygen rather than lattice oxygen, often resulting in lower activity compared to reducible supports such as CeO_2_.^32^ Apart from the choice of support, the method used to prepare the catalyst influences the catalytic performance, such as wet impregnation,^7^ precipitation,^6^ and combustion synthesis.^23^ The conventional wet impregnation is a simple and economical method that generally produces catalysts with high surface areas and well-dispersed species, although the resulting metal–support interactions are often relatively weak. In contrast, coprecipitation promotes a more homogeneous distribution of active sites and support precursors, yielding smaller crystallite sizes, stronger metal–support interactions, and improved redox characteristics, which together contribute to enhanced catalytic stability and activity.^33^ Recently, Choya et al.^34^ prepared a CeO_2_-supported catalyst for CH_4_ oxidation with six different preparation methods among catalysts synthesized by direct calcination and ammonia precipitation provided higher surface area, improved Ce^4+^ content, and better Co dispersion, which collectively enhanced redox behavior and CH_4_ oxidation activity, while other routes led to Co_3_O_4_ segregation and lower activity. These studies highlight that both the nature of the support and the synthesis method critically determine the dispersion, redox properties, and overall catalytic performance of Co_3_O_4_-based catalysts, underscoring the need to systematically evaluate these factors to design efficient CH_4_ oxidation catalysts.

Considering all the background, the present study investigates the combined effect of support nature and synthesis routes on the low-temperature oxidation performance of Co_3_O_4_-based catalysts. The novelty of this study lies in the systematic investigation of how both the synthesis route and the support nature influence the physicochemical and catalytic properties of Co-based catalysts for low-temperature CH_4_ oxidation. Unlike most previous works that focused on a single preparation method or support, this study directly compares two different synthesis methods using two different supports, while maintaining an identical metal loading. In this work, the cobalt loading was fixed at 20 wt % to eliminate the effect of metal content, and monometallic Co_3_O_4_-based catalysts were synthesized by wet impregnation and coprecipitation, using reducible and inert supports such as CeO_2_ and SiO_2_, respectively. This approach allows a direct comparison of how the preparation route and support nature govern dispersion, redox behavior, and overall catalytic performance. The findings will guide the design of optimized Co-based catalysts for efficient methane abatement under realistic operating conditions.

## Experimental Analysis

### Materials Used

The support materials for all prepared samples were CeO_2_ and SiO_2_ powder, and precursor salts used for Co_3_O_4_ were cobalt­(II) nitrate hexahydrate (Co­(NO_3_)_2_·6H_2_O). All chemicals and reactant gases were sourced from Sigma-Aldrich with a purity greater than 99% and Air Products Gulf Gas LLC, Abu Dhabi.

### Preparation of the Catalyst

All the catalysts were prepared through two different approaches, such as the wet impregnation method (WI) and coprecipitation (CP), as shown in Figure 1. In the WI method, an appropriate amount of precursor salt was dissolved in deionized water to obtain a solution corresponding to 20 wt % Co and added dropwise to a specific amount of both support (CeO_2_ and SiO_2_) and stirred for 1 h at room temperature to ensure uniform adsorption of cobalt species. The resulting mixture was slowly evaporated at 80 °C under continuous stirring, followed by drying at 110 °C overnight. The obtained dried samples were ground into fine powder and then calcined in air at 550 °C for 4 h with a heating rate of 10 °C min^–1^ to remove the volatile compound and obtain metal oxide. The catalysts prepared through this method are denoted as CoCe­(WI) and CoSi­(WI).

**Figure 1:**
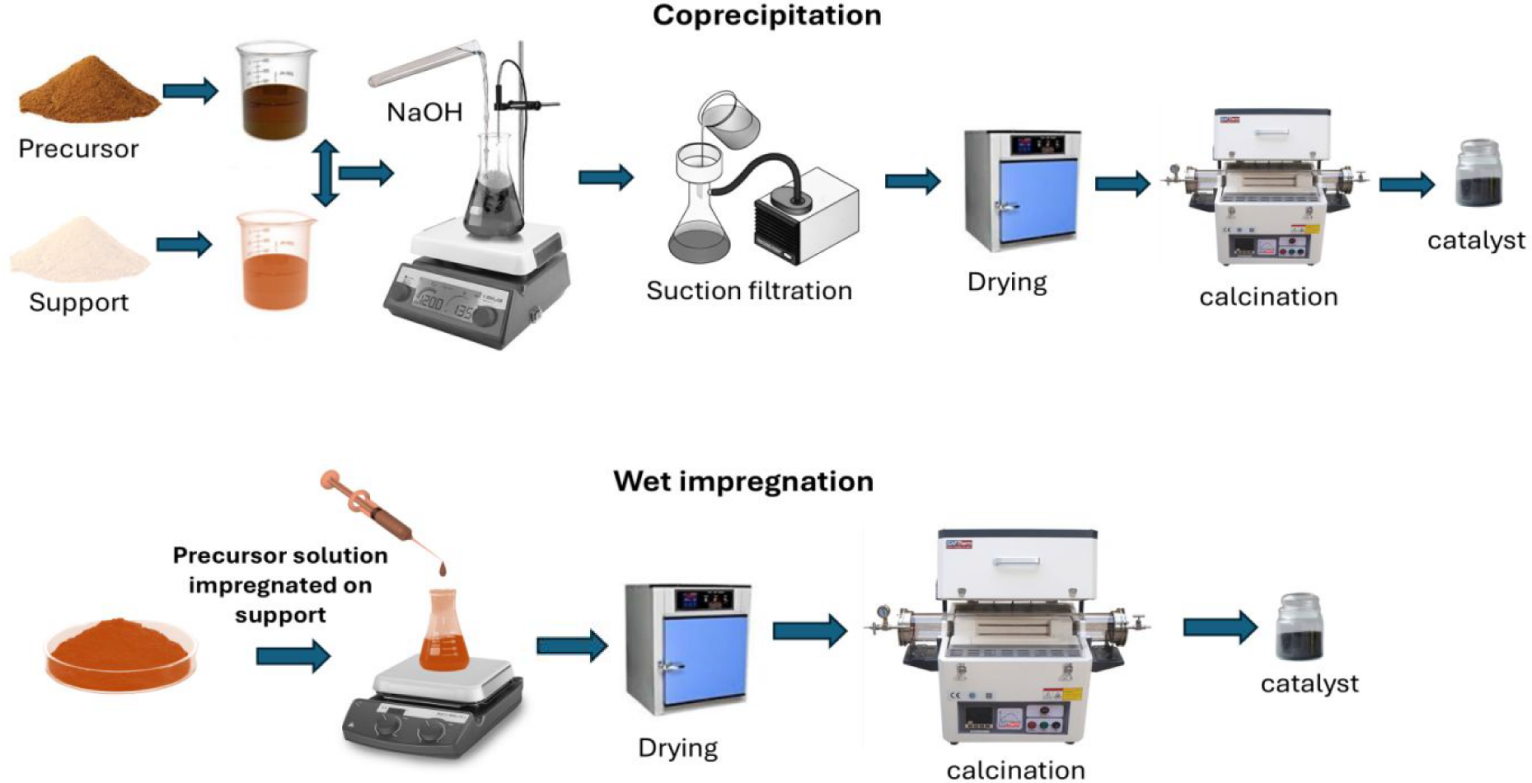
Schematic illustration of both preparation methods used to prepare the catalysts.

Similarly, for CP, the specific amounts of cobalt precursor and the corresponding support oxide were dissolved in deionized water under vigorous stirring. The solution was slowly neutralized by adding a 1 M NaOH solution dropwise until the pH reached 9, resulting in the precipitation of cobalt and support hydroxides. The precipitate was aged under stirring for 2 h, then filtered, washed thoroughly with deionized water and ethanol to remove residual ions, and dried at 110 °C overnight. At last, the obtained dried samples were ground into fine powder as mentioned above and calcined in air at 550 °C for 4 h to obtain the final form of catalysts. The catalysts prepared through this method are denoted as CoCe­(CP) and CoSi­(CP). All catalysts were synthesized with a nominal cobalt loading of 20 wt %. Semiquantitative EDS analysis was performed for the representative catalyst CoCe­(WI), and the elemental composition is provided in Table S1 (Supporting Information) to confirm the presence of cobalt.

### Characterization Analysis

Various characterization techniques were employed to investigate the structural, morphological, and surface properties of the prepared catalysts. The instruments and experimental conditions used for each method were described in our previous study.^35^ XRD analysis was performed on a Malvern Panalytical X’Pert Powder diffractometer to evaluate the crystalline structure, phase composition, and lattice parameters of the catalysts. The diffraction patterns were recorded over a 2θ range of 20°–80° using Cu Kα radiation (λ = 1.542 Å), with a scanning rate of 1° min^–1^ to ensure reliable resolution. FTIR was employed on a Jasco spectrometer (Japan) to examine functional groups and bonding interactions in the catalysts. Morphological features and elemental distributions were examined by scanning electron microscopy (SEM) equipped with an energy-dispersive spectroscopy (EDS) system, which enabled both surface imaging and mapping of the constituent elements. Further, XPS was carried out on a Thermo Scientific Nexsa G2 spectrometer to analyze the surface composition and the electronic states of the elements. Raman spectroscopy was performed on a Renishaw RM1000 instrument with a 514 nm Ar^+^ laser as the excitation source. The system provided a resolution of ±1 cm^–1^. Reducibility and oxygen mobility of the prepared catalyst were studied by H_2_-TPR and O_2_-TPD using a Micromeritics Autochem 2790 instrument. SEM and XPS analyses were performed on selected representative catalysts to interpret the effects of synthesis method and support nature on morphology and surface chemical states. SEM analysis and XPS analysis was carried out for CoCe­(WI) and CoSi­(WI) only to highlight the influence of reducible and inert supports. BET surface-area measurements were not available for this study, and the catalytic trends are therefore discussed mainly in relation to redox properties, oxygen mobility, and metal–support interactions.

### Catalytic Activity Evaluation

The catalytic performance for all prepared samples was assessed within the temperature range of 250–600 °C, under atmospheric pressure, and each measurement was taken at intervals of 50 °C. The proposed reactions were carried out in a fixed-bed quartz tubular reactor, as shown in Figure 2, which has a 12 mm inner diameter, 20 mm outer diameter, and is positioned inside a vertical electric furnace with a 200 mm uniform heating zone. For each run, 0.5 g of catalyst was loaded between two layers of quartz wool to secure the bed and to minimize temperature gradients or hot-spot formation associated with the exothermic nature of the reaction, and at each temperature step, the reactor was allowed to stabilize for 30 min before data collection. Across all experiments, complete oxidation was achieved, and no partial oxidation products, such as CO, were detected within the studied temperature window. The feed mixture consisted of 5 vol % CH_4_, 20 vol % O_2_, and helium as a balance with a total flow rate of 105 mL min^–1^ that was regulated using a mass flow controller, corresponding to a weight hourly space velocity (WHSV) of 12,600 mL g^–1^ h^–1^. The outlet of the reactor was directly connected to Shimadzu GC-2030, which analyzes the products. CH_4_ conversion was determined using the following relation,

1 $${\mathrm{CH}}_{4(\mathrm{Conversion\%})}=\left[\left({\mathrm{CH}}_{4(\mathrm{in})}{-\mathrm{CH}}_{4(\mathrm{out})}\right){/\mathrm{CH}}_{4(\mathrm{in})}\right]\times 100$$

where [CH_4_]_in_ and [CH_4_]_out_ referred as the inlet and outlet concentrations of CH_4_, respectively. The effect of WHSV on the performance of the catalyst is provided in Figure S1 (Supporting Information).

**Figure 2:**
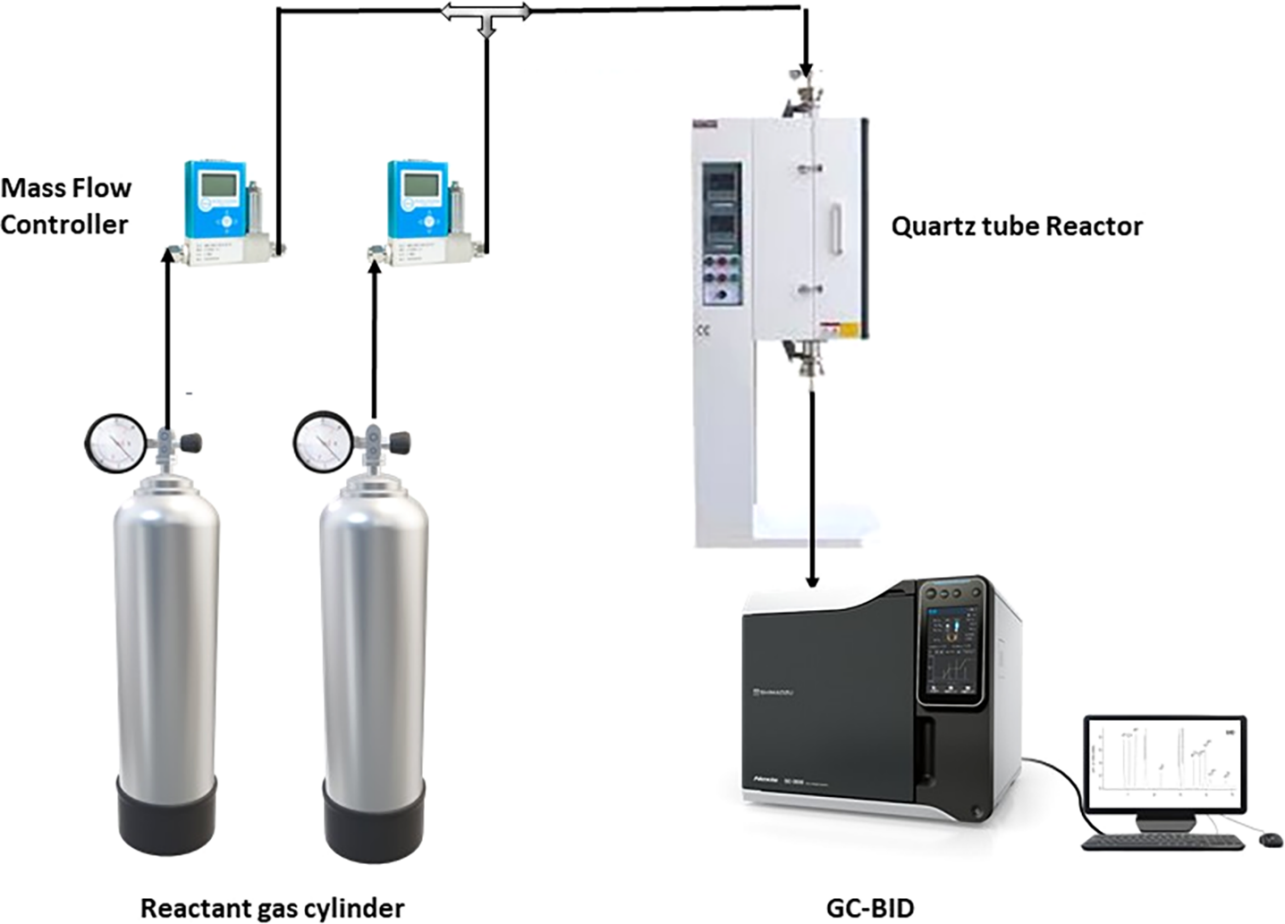
Schematic of the experimental procedure followed in this work.

## Results and Discussion

### Physicochemical Analysis of the Prepared Catalyst

#### Structural and Functional Group Analysis

The crystalline structures of all prepared catalysts and their support were examined by XRD, and the patterns recorded on the same scale display several sharp diffraction peaks within the 2θ range of 10°–80°, which can be attributed to the crystalline phases of spinel Co_3_O_4_, fluorite CeO_2_, and SiO_2_. For the bare CeO_2_ support as shown in Figure 3a, the diffraction peaks observed at 2θ values of approximately 28.7°, 33.2°, 47.7°, 56.6°, 59.3°, 69.6°, and 77.1° can be indexed to the (111), (200), (220), (311), (222), (400), and (331) planes of cubic fluorite CeO_2_ (JCPDS card no. 98-012-0143).^7^ In the case of CeO_2_-supported catalysts such as CoCe­(WI) and CoCe­(CP), all the characteristic peaks of CeO_2_ were observed on both supported catalysts, indicating that the ceria lattice was preserved after Co incorporation. In addition, weak reflections at 2θ around 36.8° and 65.2° are detected, which correspond to the (311) and (440) planes of Co_3_O_4_ (JCPDS No. 42-1467).^36,37^ The presence of these peaks confirms the formation of crystalline spinel Co_3_O_4_, particularly evident in the CoCe­(CP) sample, whereas in CoCe­(WI), the cobalt phase appears less pronounced, suggesting better dispersion of Co_3_O_4_ species within the CeO_2_ lattice. Similarly, for the SiO_2_-supported catalysts as shown in Figure 3b, the XRD pattern of the bare SiO_2_ shows broad reflections characteristic of amorphous silica with some low-intensity crystalline contributions (ICDD#00-046-1045).^38^ After the incorporation of Co, the diffraction peaks corresponding to spinel Co_3_O_4_ appear at similar 2θ positions, confirming the formation of crystalline Co_3_O_4_ phases on the silica surface. It is interesting to observe that the Co_3_O_4_ peaks in CoSi­(CP) are more intense and well-defined, as compared to the ceria-based systems, indicating a smaller crystallite size and poorer dispersion of Co_3_O_4_ on SiO_2_ support. Furthermore, to understand the impact of Co incorporation on support, the lattice parameters were calculated. The average crystallite size of the samples was estimated from the line broadening of the diffraction peaks using the Scherrer equation (eq 2), and the interplanar spacing (*d*) was calculated according to Bragg’s law (eq 3), while the lattice parameter was obtained using eq 4:

2 $$D=\frac{K\lambda }{\beta \cos\theta }$$

3 $$d=\frac{n\lambda }{2\sin\theta }$$

4 $$a=d\times \sqrt{h^{2}+k^{2}+l^{2}}$$

**Figure 3:**
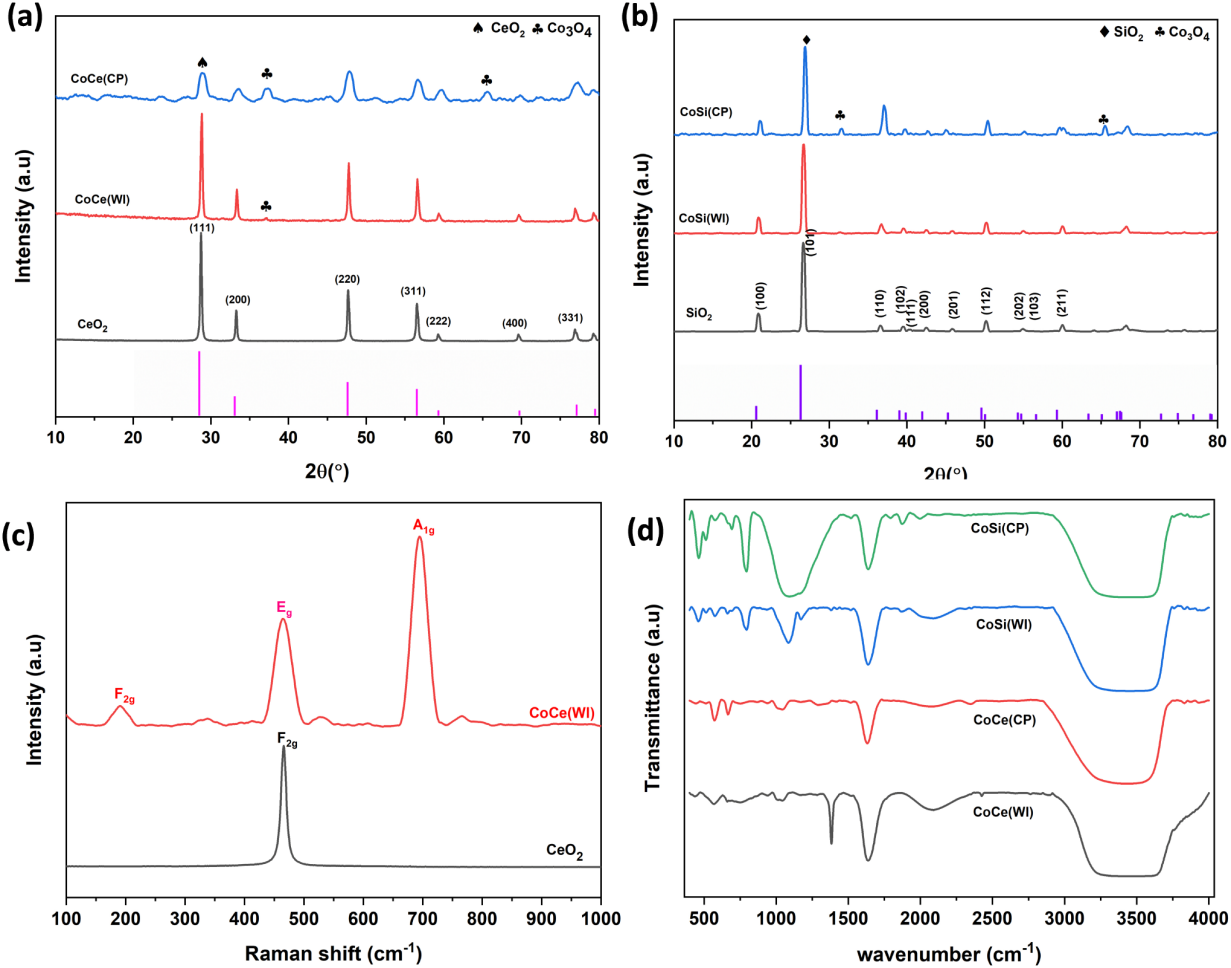
XRD patterns of prepared samples, CeO_2_ supported (a), SiO_2_-supported (b), Raman spectra of pure CeO_2_ and CoCe­(WI) (c), FTIR spectra of all prepared samples (d).

Here, “*D”* represents the average crystallite size, and “λ” is the wavelength of the incident X-ray, *“β”* is the full width at half-maximum (fwhm) of the diffraction peak, *“K”* is the Scherrer constant (shape factor), and *“θ”* is the Bragg diffraction angle. Moreover, the “*D”* corresponds to the interplanar spacing, while *“a”* denotes the lattice parameter and (*h, k, l*) represent Miller indices. The calculations were based on the most intense reflection peaks of both supported catalysts, such as the (111) plane of CeO_2_ and the (101) plane of SiO_2_, and the resulting crystallite sizes and lattice parameters are summarized in Table 1. The crystallite size for pure CeO_2_ was 32.7 nm, which slightly decreased to 30.9 nm in CoCe­(WI) and 30.8 nm in CoCe­(CP), accompanied by a small reduction in lattice constant from 5.38 to 5.35 Å which can be attributed to the incorporation of Co into the CeO_2_ lattice because dopant Co (0.65 Å) has smaller ionic radii than Ce^4+^ (0.97 Å) which facilitate the creation of oxygen vacancies.^39^ In contrast, silica-supported samples showed much smaller crystallite size around 20 nm, with the lattice constant decreasing from 4.73 Å in pure silica to 4.68 Å in CoSi­(CP). The smaller crystallite size of SiO_2_ reflects the amorphous character of the support, whereas the sharper Co_3_O_4_ peaks indicate the formation of larger crystalline particles, suggesting poorer Co dispersion and weaker catalytic performance compared to CeO_2_-supported catalysts. In this way, XRD results confirm that the fluorite structure of CeO_2_ is maintained after the incorporation of Co, although the extent of Co dispersion varies with the support and synthesis route. To further understand the insight into lattice distortions and oxygen vacancy formation, Raman spectroscopy was employed.

**Table 1:** Crystallite Size and the Lattice Constant of the Prepared Catalysts

Sample	2θ (°)	Crystallite size, *D* (nm)	Lattice constant, *a* (Å)
Pure CeO_2_	28.73	32.7	5.382
CoCe(WI)	28.80	31.0	5.368
CoCe(CP)	28.92	30.8	5.347
Pure SiO_2_	26.67	20.5	4.727
CoSi(WI)	26.70	20.4	4.722
CoSi(CP)	26.90	20.0	4.688

To further investigate the structural characteristics of the Co-based catalyst supported on CeO_2_, Raman analysis was performed only for the best-performing sample, CoCe­(WI), in comparison with pure CeO_2_ to confirm the impact of cobalt incorporation on lattice defects of the catalyst. The spectrum of pure CeO_2_, as shown in Figure 3c, shows characteristic bands centered at approximately 465 cm^–1^ corresponding to the F_2g_ vibrational modes of the fluorite lattice, which arises from the symmetric stretching of the Ce–O_8_ vibrational units, which is the prominent peak of the fluorite-type CeO_2_ structure.^40^ In addition, after the incorporation of cobalt, three prominent peaks are detected in the CoCe­(WI), which indicates the preservation of the fundamental fluorite framework of CeO_2_ along with the generation of additional lattice defects.^41^ In the CoCe­(WI) sample, the observed peaks at 191 and 465 cm^–1^ can be attributed to F_2g_ symmetry and E_g_ mode, respectively, whereas the band at 695 cm^–1^ corresponds to the A_1g_ mode.^42^ The low-frequency peak observed at 191 cm^–1^ is typically associated with vibrations of cobalt ions in tetrahedral coordination (Co^2+^), whereas the higher-frequency band near 695 cm^–1^ correlated with CoO_6_ octahedral units (Co^3+^) in the Co_3_O_4_ phase.^43^ Furthermore, all the characteristic peaks of the CoCe­(WI) catalysts appear broadened, as compared to pure CeO_2_, indicating that cobalt ions are incorporated into the CeO_2_ lattice, leading to the formation of lattice defects which create additional oxygen vacancies.^44^ These formed defect sites enhanced oxygen mobility and redox behavior, which are directly associated with the improved catalytic activity observed for the CoCe­(WI) sample.

Further, FTIR analysis was used to investigate the surface functional groups and bonding interactions present in the Co-based catalysts prepared by WI and CP methods, using CeO_2_ and SiO_2_ as supports. The analysis was carried out in the 400–4000 cm^–1^ range, and spectra of all prepared catalysts, as shown in Figure 3d, reveal characteristic absorption bands corresponding to metal–oxygen vibrations, hydroxyl groups, and surface-adsorbed species. Normally, in the low wavenumber region (500–800 cm^–1^), distinct bands are observed, which are typically assigned to metal–oxygen (Co–O, Ce–O, and Si–O) stretching vibrations in the catalyst lattice.^35^ In case of both CeO_2_-supported samples, a very weak band below 500 cm^–1^ is detected, which can be attributed to Ce–O vibrations, confirming the preservation of the fluorite-type structure of ceria. Additionally, the two other peaks at 572 and 676 cm^–1^, which are more prominent in CoCe­(CP), are assigned to the Co–O vibration absorption characteristic of spinel Co_3_O_4_.^7^ Meanwhile, in SiO_2_-supported catalysts, a strong band around 1080–1100 cm^–1^ corresponds to asymmetric stretching of Si–O–Si linkages, while the peaks near 786 cm^–1^ and 455 cm^–1^ are assigned to symmetric stretching and bending vibrations of Si–O bonds, respectively.^45^ These results indicate the structural integrity of the silica framework even after cobalt deposition. Furthermore, the presence of weak bands near 1400–1450 cm^–1^ can be assigned to carbonate-like species originating from atmospheric CO_2_ adsorption on basic sites of the oxide support, which is more prominent in CeO_2_-supported catalysts, due to its oxygen vacancy-rich surface.^46^ Apart from this, all catalysts exhibit broad absorption bands in the region of 3200–3600 cm^–1^, which are ascribed to the O–H stretching vibrations of surface hydroxyl groups and adsorbed water molecules.^47^ Moreover, the corresponding bending vibrations of molecular water appear around 1620–1640 cm^–1^.^48^ It is interesting to observe that CoCe­(WI) displays a more intense and broader O–H stretching band compared to CoCe­(CP), suggesting a higher concentration of surface hydroxyl groups and adsorbed moisture, which may influence redox behavior and catalytic activity. These observations confirm other functional groups and the bonding characteristics of Co with CeO_2_ and SiO_2_ supports without significant changes to the supported oxide structures.

### Morphological Analysis

To investigate the surface morphology of the catalysts, SEM analysis was carried out for both CoCe (WI) and CoSi­(WI), as shown in Figure 4. The SEM image of CoCe (WI) (Figure 4a) reveals an irregular morphology composed of interconnected particles, which is typical of ceria-based oxides^49^ and is expected to provide a high density of active sites, which is beneficial for catalytic oxidation reactions. In addition, the elemental distribution was further analyzed by EDS mapping, which confirmed that Ce, Co, and O atoms are homogeneously distributed across the catalyst surface, indicating effective dispersion of cobalt over the ceria support without any detectable impurities.In contrast, the SEM image of CoSi­(WI) in Figure 4b shows a relatively different morphology, where Co_3_O_4_ particles appear more aggregated and less uniformly distributed over the SiO_2_ support. Further, the corresponding EDS mapping confirms the presence of Si, Co, and O elements across the surface; however, the cobalt distribution appears less homogeneous compared to CoCe­(WI), indicating weaker metal–support interaction. Moreover, the point analysis for both samples as shown in Figure S2 (Supporting Information, SM) confirms that all elements are present at their characteristic energy values and all metal contents in CoCe­(WI) and CoSi­(WI), with their atom and mass % values, are presented in Tables S1 and S2 (Supporting Information, SM), where the composition is consistent with the expected Co–Ce–O and Co–Si–O frameworks and reflects the successful incorporation of cobalt into the support lattice. The uniform distribution of cobalt, along with the absence of large Co-rich agglomerates, suggests stronger metal–support interaction in CoCe­(WI) compared to CoSi­(WI), which is known to enhance oxygen vacancy formation and redox behavior. These characteristics are consistent with the Raman results, where defect-induced modes confirmed the introduction of lattice distortions upon cobalt incorporation.

**Figure 4:**
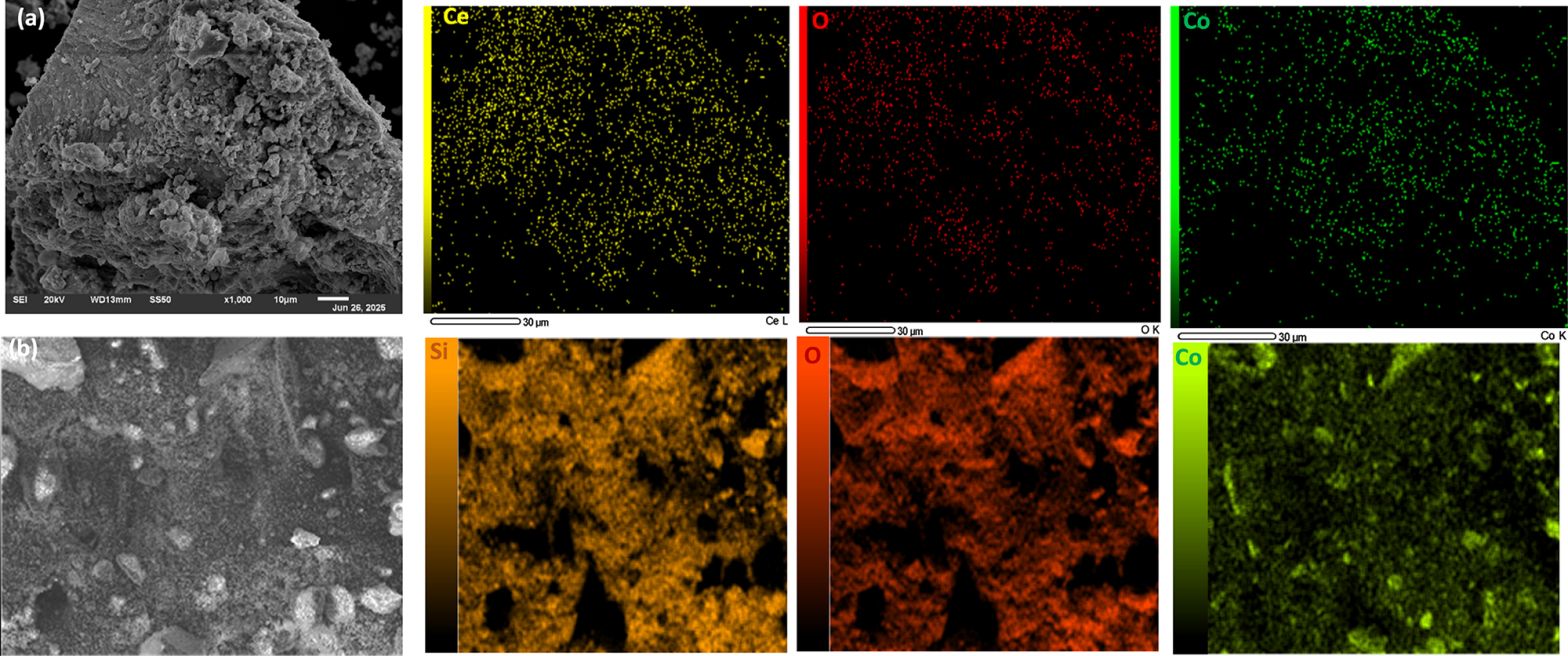
SEM images of CoCe­(WI) (a) and CoSi­(WI) (b) catalysts, along with their corresponding EDS elemental mapping.

### Surface Analysis

XPS was employed to investigate the surface chemical states of the catalysts, focusing only on CoCe (WI) and CoSi­(WI), to explain the influence of the support on the electronic environment of the doped metal. The XPS survey spectra of both catalyst CoCe­(WI) and CoSi­(WI), as shown in Figure 5, exhibit the characteristic signals corresponding to Co 2p, O 1s, and the respective support elements, Ce 3d and Si 2p, which confirm the successful dispersion of doped Co species on both CeO_2_ and SiO_2_ surfaces. The Ce 3d spectra of CoCe­(WI) as shown in Figure S3, in which binding energy features are denoted as U and V, corresponding to the Ce 3d_3/2_ (higher binding energy) and Ce 3d_5/2_ (lower binding energy) spin–orbit components, respectively.^50^ The Ce 3d spectrum detected peaks at 882.3, 888.7, 898.2, 900.8, 906.4, and 916.3 eV, which are assigned to Ce^4+^, while additional features at 880.5, 885.3, and 903.7 eV correspond to Ce^3+^. The coexistence of both Ce^3+^ and Ce^4+^ oxidation states confirms the presence of oxygen vacancies together with fully oxidized ceria, highlighting the redox-active nature of the support, which enhances oxygen mobility and strengthens the cobalt–support interaction,^51^ thereby promoting CH_4_ oxidation. On the other hand, the Si 2p spectrum of CoSi­(WI), as shown in Figure S4, exhibits a single peak, characteristic of Si^4+^ in SiO_2_,^52^ which confirms that SiO_2_ as a support remains in a fully oxidized, chemically stable, and nonreducible state, lacking the ability to create oxygen vacancies or participate in redox processes.

**Figure 5:**
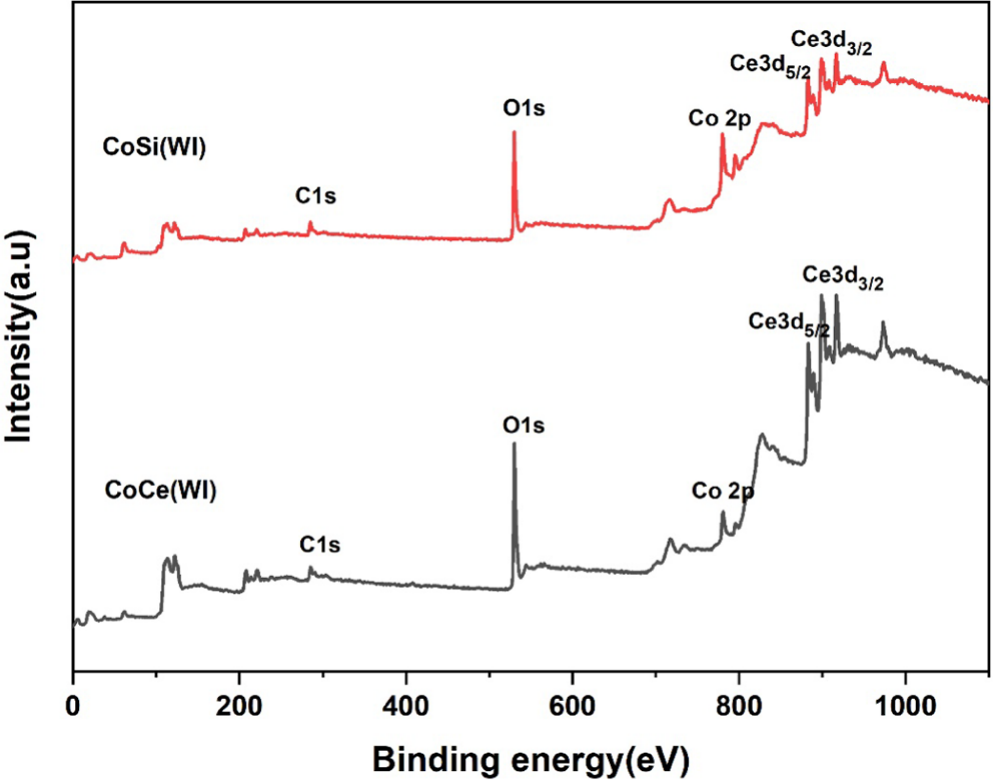
XPS survey of catalyst CoCe­(WI) and CoSi­(WI).

The Co 2p spectra of both catalyst CoCe­(WI) and CoSi­(WI) are shown in Figure 6a, in which both catalysts exhibit two main peaks corresponding to Co 2p_3/2_ and Co 2p_1/2_, between 778 eV–795 eV, along with associated satellite peak, confirming the presence of Co in mixed oxidation states. After deconvoluting the Co 2p spectra, as shown in Figure 6b, reveals contributions from both Co^2+^ and Co^3+^ oxidation states. The Co 2p_3/2_ peak of CeO_2_-supported catalyst is divided into the two components located at 779.39 and 780.68 eV, corresponding to Co^3+^ and Co^2+^ species, respectively.^53^ Similarly, the Co 2p_1/2_ peak is split into two contributions at 794.43 eV (Co^3+^) and 795.98 eV (Co^2+^).^54^ In the case of CoSi­(WI), the Co 2p_3/2_ peak shows two components at 779.04 eV (Co^3+^) and 780.35 eV (Co^2+^), while the Co 2p_1/2_ peak consists of two contributions at 794.11 eV (Co^3+^) and 795.73 eV (Co^2+^), as shown in Table 2. In both cases satellite peak of the cobalt spectra was dominated by an intense signal centered around 785.05 eV. The quantitative XPS analysis reveals distinct differences in the surface chemistry of CoCe­(WI) and CoSi­(WI). The area ratios of Co^2+^ in tetrahedral coordination and Co^3+^ in the octahedral coordination (A_Tetrahedral_/A_Octahedral_), indicating the surface of Co^2+^/Co^3+^, were calculated as 2.63 for CoCe­(WI) and 2.5 for CoSi­(WI), indicating that both catalysts are dominated by Co^2+^ species with only minor variations in the distribution of oxidation states. Similar observations have been reported in previous studies,^21^ where a higher A_Tetrahedral_/A_Octahedral_ ratio was correlated with improved catalytic activity.

**Table 2:** XPS Binding Energies (eV) and Surface Ratios of CoCe­(WI) and CoSi­(WI)

Catalyst	Ce 3d (eV)	Co 2p_3/2_ (eV)	Co 2p_1/2_ (eV)	Si 2p (eV)	O 1s (eV)	Co^2+^/Co^3+^	O_ad_/O_latt_
CoCe(WI)	Ce^3+^: 880.5, 885.3, 903.7 Ce^4+^: 882.3–916.3	779.39 (Co^3+^), 780.68 (Co^2+^)	794.43 (Co^3+^), 795.98 (Co^2+^)	–	529.0 (O_latt_), 531.2 (O_ad_)	2.63	1.14
CoSi(WI)	–	779.04 (Co^3+^), 780.35 (Co^2+^)	794.11 (Co^3+^), 795.73 (Co^2+^)	106	529.0 (O_latt_), 531.0 (O_ad_)	2.50	0.84

**Figure 6:**
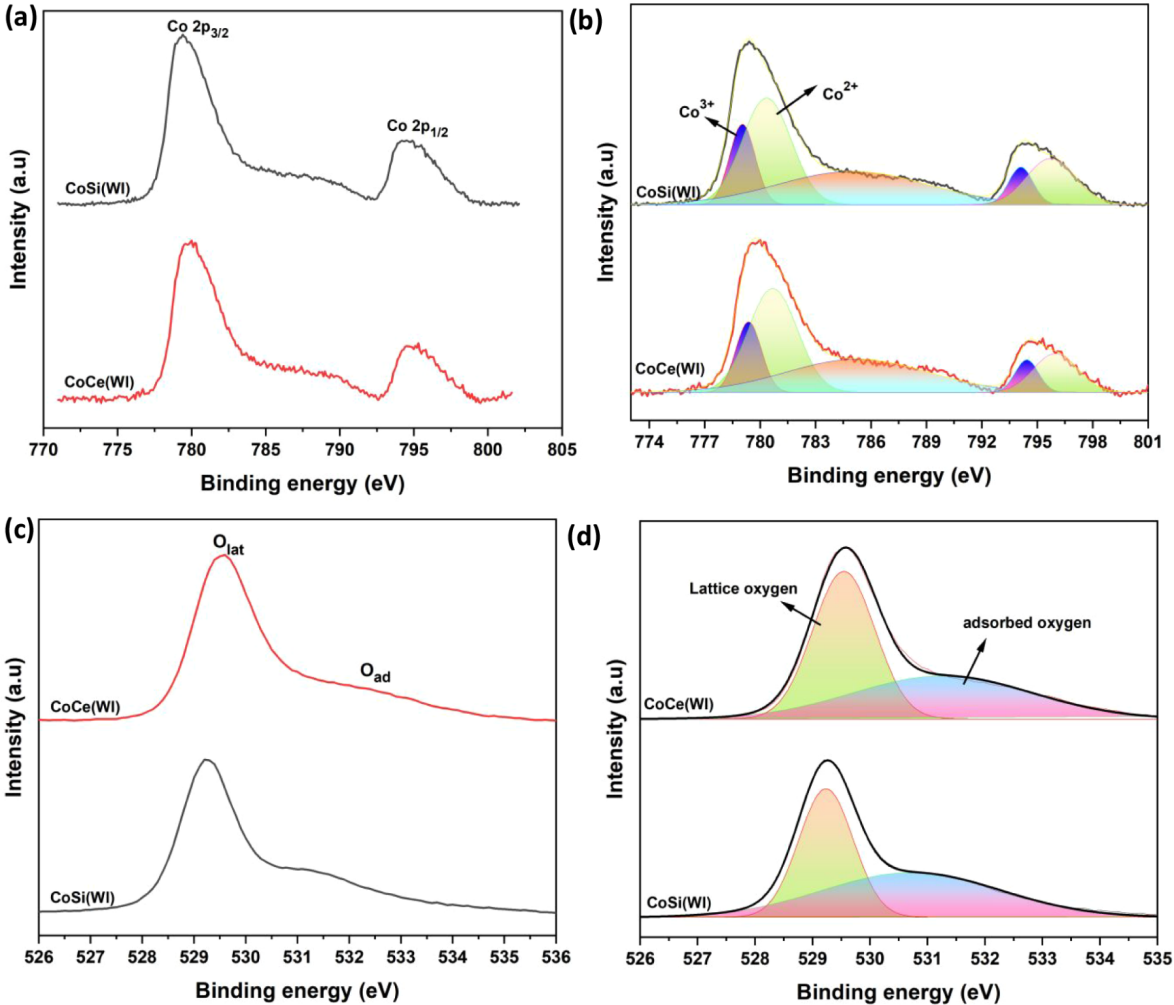
XPS spectra of experimental Co 2p (a); fitted Co 2p (b); experimental O 1s (c); fitted O 1s (d).

The O 1s spectra and their deconvoluted peaks of both catalysts are depicted in Figure 6c–d. The two main peaks appearing in the 529–530 eV and 530.7–531.5 eV range are attributed to lattice oxygen, denoted as O_latt._ and surface-adsorbed oxygen species (O_ad_), respectively.^54^ The ratio of O_ad_/O_latt_ were determined to be 1.14 for CoCe­(WI) and 0.84 for CoSi­(WI), suggesting that the CeO_2_-supported catalyst contains a larger fraction of surface-adsorbed oxygen species relative to lattice oxygen, which are widely recognized as active sites for low-temperature CH_4_ oxidation.^55^ In this way, it clearly demonstrates that the support employed a strong influence on the surface chemical states of cobalt and oxygen. Although the O 1s spectra of CoSi­(WI) display a more visible shoulder assigned to adsorbed oxygen, quantitative fitting of the peaks provides a clearer comparison. This difference is attributed to the Ce^4+^/Ce^3+^ redox cycle of CeO_2_, which promotes the continuous formation and replenishment of oxygen vacancies even when the spectral distinction is not visually prominent. Therefore, the higher oxygen-adsorption capacity of CoCe­(WI) arises from its intrinsic redox flexibility rather than the intensity of the shoulder feature alone. Further, the CoCe­(WI) exhibits enhanced Co^2+^ content and a higher proportion of adsorbed oxygen species due to the redox-active nature of CeO_2_ support. While the SiO_2_-supported catalyst, CoSi­(WI), shows weaker metal–support interaction, lower Co^2+^ concentration, and limited oxygen activation capability, which can be attributed to its lower catalytic activity compared to CoCe­(WI).

### Redox and Surface Oxygen Analysis

The redox properties of all prepared samples were analyzed by H_2_-TPR, which clearly highlights the influence of both the support and the synthesis method on the reducibility of the catalyst. Previous studies have shown that pristine CeO_2_ exhibits reduction peaks at typically higher temperatures, which is attributed to the removal of surface oxygen species that generate oxygen vacancies and promote the migration of lattice oxygen from the bulk to the surface of CeO_2_.^56^ In contrast, SiO_2_ is generally considered a redox-inert support, and previous studies have reported the absence of noticeable reduction peaks for pure SiO_2_.^57^ To confirm this, the H_2_-TPR profiles of bare SiO_2_, bare CeO_2_, and Co_3_O_4_ were also measured and are presented in the Figure S5. As expected, SiO_2_ showed no detectable reduction peaks, confirming its redox-inert nature, while CeO_2_ exhibited a broad surface-reduction feature centered around 460 °C, associated with the Ce^4+^ → Ce^3+^ transition, while Co_3_O_4_ displayed a sharp reduction peak near 380 °C corresponding to the Co^3+^ → Co^2+^ step. Further, the integrated peak data, including the maximum peak temperature and the corresponding volume of H_2_ consumed, as presented in Table 3. As shown in Figure 7, among the investigated samples, CoCe­(WI) exhibits three distinct reduction peaks, including two midtemperature peaks between 200–300 °C and a more intense peak centered at around 357.4 °C. These features are characteristic of highly dispersed Co_3_O_4_ that strongly interacts with CeO_2_ support. The low-temperature peaks are associated with the reduction of surface Co^3+^ species to Co^2+^, involving the removal of surface oxygen, while the higher-temperature peak corresponds to the further reduction of strongly bound Co^2+^ to metallic Co, associated with bulk oxygen removal.^6,58^ Notably, lower-temperature peaks are associated with more reactive cobalt species, which promote the catalytic activity.^59^ It is interesting to observe that CoCe­(CP) displays only a weak and broad reduction peak near 393.5 °C, pointing to lower Co dispersion and weaker Co–CeO_2_ interaction compared to CoCe­(WI), which limits the availability of active oxygen and thereby reduces catalytic activity. The silica-supported samples exhibit even poorer reducibility compared to their CeO_2_ counterpart. The catalyst prepared through the wet impregnation method, named CoSi­(WI), at least shows a broad peak near 357 °C, while other catalysts prepared through different methods, named CoSi­(CP) presents only a very faint peak, indicative of weak metal–support interaction and minimal oxygen participation.^60^ Interestingly, catalysts prepared using the CP method exhibit small peaks at lower temperatures compared to those prepared by WI method, suggesting that WI method can provide a stronger interaction between Co_2_O_3_ and support oxide than the CP method. Furthermore, the total H_2_ consumption values confirm this trend, with CoCe­(WI) exhibiting the highest uptake (0.8693 mL g^–1^ STP) compared to other samples, indicating a greater number of reducible oxygen species and a higher concentration of oxygen vacancies that directly contribute to its superior CH_4_ oxidation activity. These observations are in line with earlier studies^33,61^ showing that CP and WI methods exhibits different temperature peaks for a prepared catalyst. Hence, the superior CH_4_ oxidation activity of CoCe­(WI) can be attributed to its improved reducibility, strong Co–CeO_2_ synergy, and enhanced oxygen mobility, which together provide the reactive oxygen species required for complete combustion.

**Table 3:** Peak Position and H_2_ Uptake of H_2_ TPR Profiles in a Prepared Catalyst

	Peak Position(°C)		
Catalysts	I	II	Total H_2_ consumption (mL/g STP)
CoCe(WI)	299.3	357.4	0.8693
CoCe(CP)	336.3	393.5	0.1189
CoSi(WI)	309.6	359.8	0.3908
CoSi(CP)	362.1	527.1	0.0773

**Figure 7:**
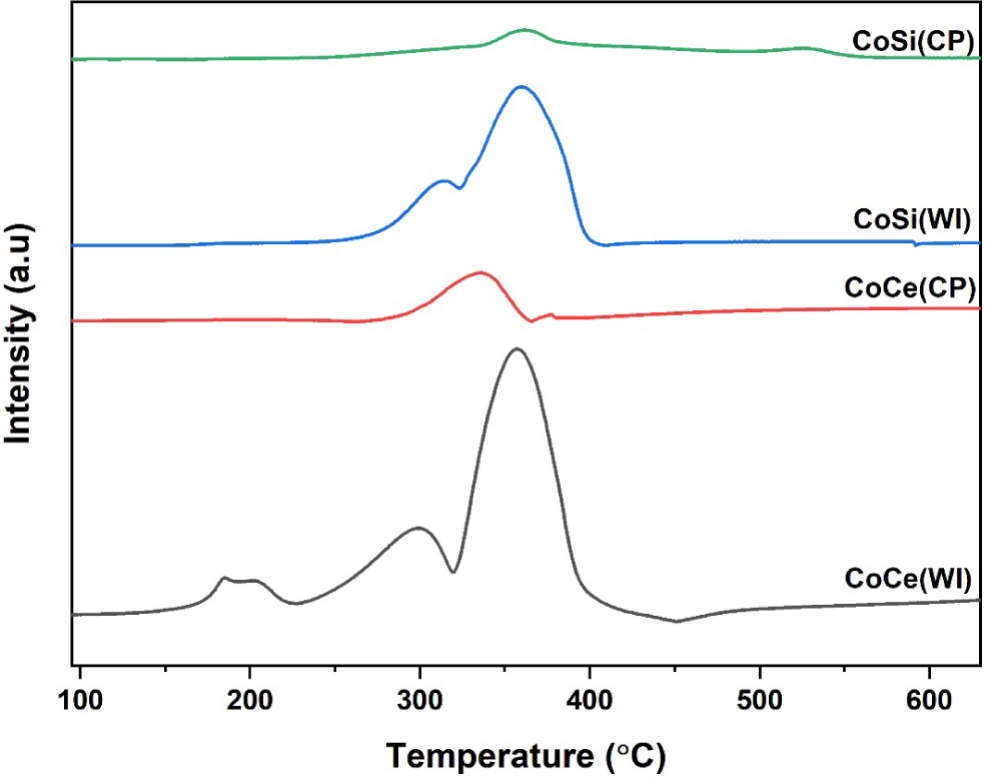
H_2_ TPR profile of all prepared catalysts in a flow of 5% H_2_ balanced in He.

In the CH_4_ oxidation process, lattice oxygen from the oxide surface participates in the conversion of CHx species, producing oxygen vacancies that are replaced by gaseous oxygen. Meanwhile, both the replaced adsorbed oxygen and the surface lattice oxygen are important in oxidation catalysis, as they provide active sites for oxygen activation and promote the oxidation of intermediates on the catalyst surface.^62^ Further, to investigate the nature, strength, and mobility of oxygen species present on the catalyst surface, O_2_-TPD was carried out as shown in Figure 8. Typically, in this analysis, O_2_ desorption peaks were observed below 300 °C, which is attributed to weakly adsorbed surface oxygen species (O_2_^–^/O^–^),^63^ while desorption in the 300–500 °C range corresponds to more strongly bound surface lattice oxygen, and peaks above 500 °C are generally associated with bulk lattice oxygen.^64^ In all tested catalysts, CoCe­(WI) exhibits the most pronounced and well-resolved desorption features, with oxygen release extending from low to midtemperature ranges, which indicates the presence of highly mobile surface oxygen species on the catalyst surface. For comparison, the O_2_-TPD profiles of CeO_2_ and SiO_2_ were also examined and are provided in the Figure S6. The TPD profile of CeO_2_ displayed a broad desorption feature between 350–500 °C, corresponding to the release of weakly bound surface oxygen species associated with its Ce^4+^/Ce^3+^ redox pair, whereas SiO_2_ showed no noticeable desorption peak, confirming its nonreducible and oxygen-inert nature. The oxygen mobility is consistent with the strong Co and CeO_2_ interaction observed in the H_2_-TPR results, where CeO_2_ provides a dynamic oxygen reservoir through its Ce^4+^/Ce^3+^ redox couple.^65,66^ The presence of these reactive oxygen species is known to be crucial for oxidation reactions, as they promote the generation of oxygen vacancies, which enhance the catalytic activity for CH_4_ combustion.^67^ On the other hand, CoCe­(CP) shows a much weaker desorption peak, centered around the midtemperature region (∼350 °C), suggesting limited oxygen availability and weaker Co–CeO_2_ synergy compared to CoCe­(WI). A similar trend is observed for the silica-supported catalyst, where both CoSi­(WI) and CoSi­(CP) display weak desorption peaks. The observed differences can be attributed to the synthesis method, where WI favors better dispersion of cobalt species and stronger Co and CeO_2_ interactions, while coprecipitation often results in lower dispersion and partially blocked active sites.^68^ Overall, the O_2_-TPD results confirm that the superior performance of CoCe­(WI) arises from its abundant and readily available oxygen species, enabled by strong metal–support interactions and the inherent oxygen storage/release capacity of CeO_2_. The influence of preparation method is also evident in a previous study,^69^ but in our case, wet impregnation consistently yields catalysts with higher oxygen mobility than coprecipitation.

**Figure 8:**
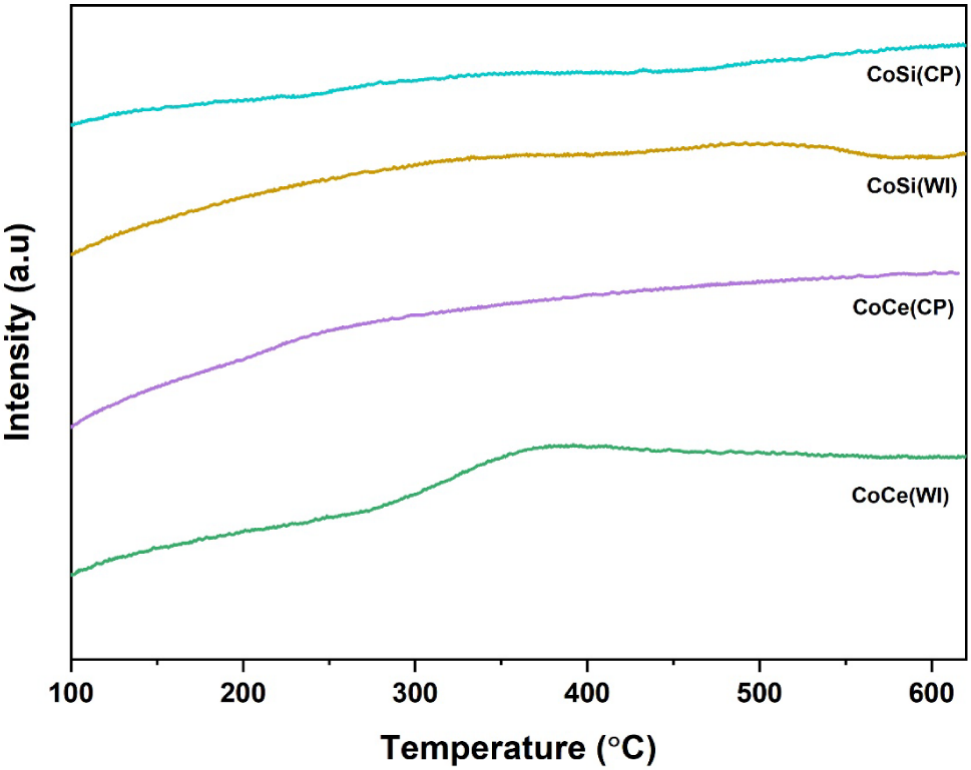
O_2_ TPD profile of all prepared samples.

### Activity Measurements of the Prepared Catalyst

#### Catalytic Effectiveness of Prepared Catalyst

To investigate the effectiveness of all prepared catalysts for CH_4_ conversion at low temperature, the catalytic activity of all prepared catalysts was studied under the same reaction conditions, and all samples demonstrated typical light-off behavior, where CH_4_ conversion increases significantly with temperature across the 250–600 °C range. At lower temperatures, CH_4_ conversion remained minimal for all catalysts, reflecting the intrinsic difficulty of activating the strong C–H bonds of CH_4_. As the temperature increased, all catalysts observed a clear rise in conversion, confirming the thermally activated nature of the catalytic oxidation process, which is constraint with the previous study.^6,69^ It is important to note that all catalysts exhibited complete selectivity toward CO_2_ across the entire temperature range, with no detectable formation of side products such as CO at any point. The light-off curves of CH_4_ conversion for all the prepared catalysts are depicted in Figure 9, where a catalyst prepared using the WI method, CoCe­(WI), showed the highest activity, reaching 91% conversion at 600 °C. On the other hand, the catalyst synthesized by the CP method, CoCe­(CP), exhibited the lowest catalytic activity among all the prepared catalysts, with CH_4_ conversion reaching only about 62% at the highest reaction temperature. Meanwhile, the catalysts supported on SiO_2_, such as CoSi­(WI) and CoSi­(CP), showed moderate catalytic activity, achieving maximum conversions of 68% and 67%, respectively. For comparison, catalytic activity was carried out using bare CeO_2_, SiO_2_, and unsupported Co_3_O_4_ under identical conditions. The bare supports (CeO_2_ and SiO_2_) showed no measurable CH_4_ conversion (<1%) throughout 250–600 °C, whereas Co_3_O_4_ exhibited gradual activity reaching about 45% conversion at 600 °C. To better evaluate the low-temperature activity, the light-off temperature (T_50_), defined as the temperature at which 50% CH_4_ conversion is achieved, was determined from the conversion curves. The CoCe (WI) catalyst shows the lowest T_50_ value of approximately 430 °C, followed by CoSi (CP) (478 °C), followed by CoSi­(WI) and CoCe­(CP) with 530 and 550 °C, respectively. In contrast, unsupported Co_3_O_4_ does not reach 50% conversion within the studied temperature range. These results confirm the superior low-temperature performance of the CeO_2_-supported catalyst prepared by wet impregnation. These results confirm that the catalytic activity mainly arises from the active Co species and is further enhanced when Co is dispersed on the reducible CeO_2_ support. The enhanced catalytic performance of CoCe­(WI) is primarily attributed to the high oxygen storage capacity and redox flexibility of CeO_2_, which enhances oxygen mobility that promotes combustion at a lower temperature.^70,71^ It is important to note that the CoCe­(CP) catalyst, despite being supported on reducible CeO_2_, showed a low conversion of around 65%, which may be related to less effective cobalt dispersion and weaker metal–support interaction, as suggested by its weaker reducibility behavior. Additionally, from physicochemical analysis, it is confirmed that the WI method promoted better Co dispersion and stronger metal–support interactions compared to the CP method, thereby increasing the number of active surface sites for CH_4_ activation.^1^ This interpretation is supported by XRD results, where CoCe­(CP) exhibits more intense Co_3_O_4_ peaks than CoCe­(WI), indicating larger crystallites and poorer Co dispersion. In addition, the H_2_-TPR profile of CoCe­(CP) shows a single weak reduction peak with much lower total H_2_ consumption, confirming fewer reducible Co species. These results together confirm the restricted accessibility of active sites and explain the weaker reducibility and lower CH_4_ conversion observed for CoCe­(CP). Interestingly, the SiO_2_-supported catalysts named CoSi­(WI) and CoSi­(CP) showed intermediate activity, reaching around 67% CH_4_ conversion at 600 °C. Although SiO_2_ is nonreducible, its high surface area and inert nature aid in dispersing Co_3_O_4_ nanoparticles, especially in the CP method, which may explain why CoSi (CP) slightly outperforms CoSi (WI). However, in SiO_2_-supported catalysts, activity mostly depends on surface-adsorbed oxygen species rather than lattice oxygen, due to the absence of redox function, which limits oxygen mobility and ultimately activity.^12^ In conclusion, the trend underscores that the catalyst preparation method significantly affects the dispersion, crystallinity, and reducibility of the active phase, especially when paired with supports that either aid or hinder oxygen mobility.^34,72^

**Figure 9:**
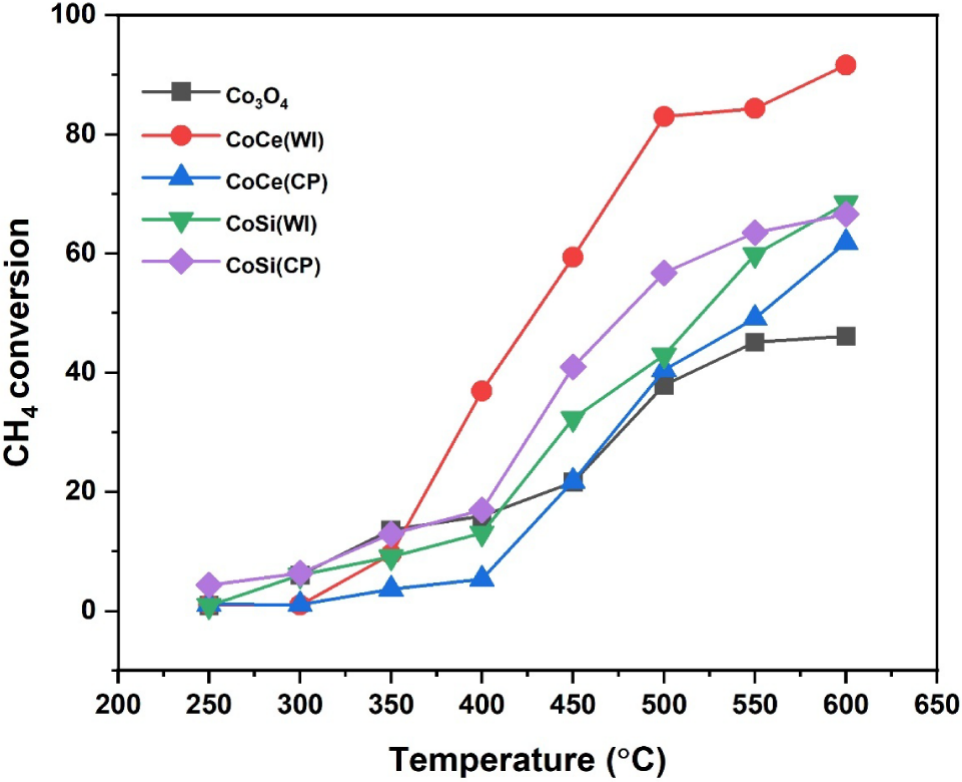
Light-off curves of all prepared catalysts in the 250–600 °C temperature range, under reaction conditions (5 vol % CH_4_, 20 vol % O_2_ balanced with He, WHSV 12600 cm^3^ g^–1^ h^–1^).

In the literature, several studies have reported the oxidation of CH_4_ over Co_3_O_4_-based catalysts supported on different oxides; however, the activity and stability are highly dependent on the choice of support, preparation route, and Co–support interaction, as shown in Table 4. For instance, Dou et al.^6^ prepared a Co_3_O_4_/CeO_2_ nanocomposite by the precipitation method, which achieved an 88% CH_4_ conversion at 600 °C, while Darda et al.^69^ achieved 84% conversion for Co_3_O_4_ supported on CeO_2_ synthesized via a sol–gel route. Similarly, Co_3_O_4_ supported on different metal oxides, such as γ-Al_2_O_3_ ^23^ and SnO_2_ ^20^ exhibited moderate activities of 75% and 70% at 600 °C, respectively, which can be attributed to their limited redox capacity and weaker metal–support interaction. Wu et al.^73^ reported 90% conversion for Co_3_O_4_ particles grown on nanocrystalline CeO_2_, while Choya et al.^34^ obtained around 87% conversion using CeO_2_ supports prepared by different synthesis routes. Compared with previously reported catalysts, although reaction parameters vary among different studies, the results indicate the excellent activity of the catalyst synthesized in this work. It should be noted that direct comparison of maximum CH_4_ conversion values across different studies is limited due to variations in feed composition, space velocity, and reaction conditions. The CoCe­(WI) catalyst developed in this work achieved a 91% CH_4_ conversion at 600 °C, indicating competitive performance among non-noble-metal catalysts tested under similar operating conditions. The effect of WHSV on the performance of the CoCe­(WI) catalyst is provided in the Supporting Information.

**Table 4:** Comparative Performance of Co_3_O_4_-Based Catalysts for Low-Temperature CH_4_ Oxidation

Catalyst composition	Synthesis method	Reaction conditions	Space velocity	Conversion	Reference
CoCe	Wet impregnation	5 vol % CH_4_, 20% O_2_, He balance	12,600 cm^3^ g^–1^ h^–1^	T_50_ = 430 °C	This work
Co_3_O_4_/CeO_2_ (nanocomposite)	Precipitation	10 vol % CH_4_, Pure O_2_	18000 ml g^–1^ h^–1^	T_50_ = 475 °C	^6^
Co_3_O_4_/CeO_2_ (SMO-type support)	Sol–gel	0.5 vol % CH_4_ and 10 vol % O_2_, balanced with He	40,000 h^–1^	T_50_ = 520 °C	^69^
Co_3_O_4_/γ-Al_2_O_3_	Impregnation	0.2 vol % CH_4_, 10 vol % O_2_, and N_2_ as the balance gas	36000 mL g^–1^ h^–1^	T_50_ = 584 °C	^23^
Co_3_O_4_/γ-Al_2_O_3_	Combustion synthesis	0.2 vol % CH_4_, 10 vol % O_2_, and N_2_ as the balance gas	36000 mL g^–1^ h^–1^	T_50_ = 423 °C	^23^
Co_3_O_4_/ZrO_2_	Precipitation	0.5% CH_4_, 8.0% O_2_ and 91.5% N_2_	12000 mL g^–1^ h^–1^	T_90_ = 335 °C	^21^
Co_3_O_4_/CeO_2_ (support by different routes)	Wet impregnation	1% CH_4_, 10% O_2_, 89% N_2_	60 000 h^–1^	T_50_ = 435 °C	^34^

### Stability Test and Analysis of Spent Catalysts

To study the stability of the prepared catalyst, the long-term stability test of the best-performing catalyst, CoCe­(WI), was performed at 550 °C for 12 h as a representative high-conversion temperature that allows evaluation of catalyst durability under sustained reaction conditions without excessive thermal stress. As shown in Figure 10a, the CH_4_ conversion initially measured at approximately 74% gradually decreased to about 59% after 12 h of reaction time, indicating a moderate but expected decline in activity over time. The observed deactivation can be attributed to partial surface sintering of Co_3_O_4_ particles and a gradual reduction in the number of active surface oxygen species during prolonged exposure to the reaction condition.^74^ Similar time-dependent decreases in conversion have been widely reported for Co- and Ce-based catalysts under lean-CH_4_ oxidation, where thermal restructuring or blocking of active sites by carbonate species leads to slight performance loss.^7,28^ Moreover, the catalyst retained more than 80% of its initial activity after 12 h, demonstrating good structural and redox stability under continuous reaction conditions. Furthermore, to understand the cause for the small deactivation, TGA analysis of the spent catalyst was performed under an O_2_ environment with a 10 °C min^–1^ ramp. As shown in Figure 10b, the weight loss of less than 0.7% up to 600 °C indicates negligible carbon deposition after reaction. The initial minor loss below 150 °C, which can be attributed to the removal of surface moisture or weakly bound species, while no significant weight change was observed beyond 200 °C, which confirmed that the catalyst surface remained clean and stable. Since postreaction structural characterization, such as XRD or SEM, was not performed, the decrease in activity is therefore attributed to possible surface sintering of Co_3_O_4_ species and/or gradual depletion of reactive surface oxygen species during long-term operation. This explanation is proposed as a reasonable hypothesis rather than a definitive conclusion.

**Figure 10:**
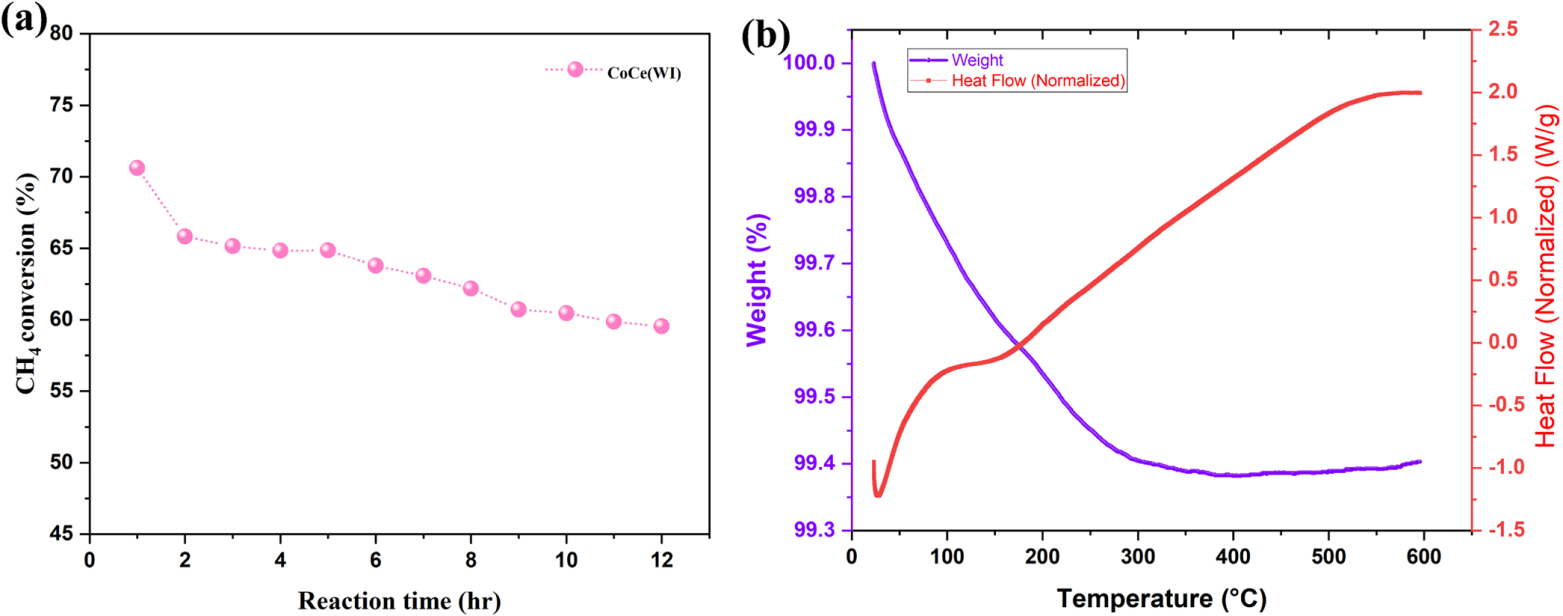
Stability test for best performing catalysts at 550 °C under reaction conditions (a), TGA analysis of spent catalyst (b).

### Correlation between Physicochemical Analysis and Catalytic Activity

The catalytic activity observed for the prepared catalysts can be directly correlated to their physicochemical characteristics. The catalysts, having been supported on CeO_2_, prepared by the wet impregnation method, CoCe demonstrated the highest conversion at low temperatures among all tested catalysts, achieving a 91% conversion value of 600 °C. This significant improvement in the activity of prepared catalysts can be properly explained by their physicochemical analysis. In which XRD confirmed that CeO_2_-supported catalysts maintained the fluorite structure even after incorporation of doped metal Co, with a slight reduction in crystallite size and lattice parameter, which indicates the substitution of smaller Co ions into the CeO_2_ lattice, which facilitated the formation of oxygen vacancies. Further, these defects enhance oxygen mobility and provide additional active sites, which explains the higher activity of CoCe­(WI). Raman spectra of CoCe­(WI) further confirmed this, as defect-related bands were detected alongside the F_2g_ mode of CeO_2_, indicating lattice distortion and oxygen vacancy generation, which improved the catalytic activity.^75^ Further, the FTIR analysis confirmed the incorporation of doped metal on different supports by revealing only characteristic bonds of Co and the support element. Afterward, SEM-EDS mapping of CoCe­(WI) confirmed uniform Co distribution on the CeO_2_ surface, consistent with XRD evidence of smaller crystallite size and stronger metal–support interaction. Together, these results demonstrate that CoCe­(WI) provides better Co dispersion and defect formation than its SiO_2_-based counterparts.

XPS analysis exhibited distinct differences between the supports that correlate with activity. In CoCe­(WI), the coexistence of Ce^3+^ and Ce^4+^ confirmed the redox cycle that stabilizes Co^3+^ species and supplies active oxygen, which directly promotes methane oxidation. Although CoSi­(WI) showed a slightly higher Co^3+^/Co^2+^ ratio, its inert SiO_2_ support lacked redox flexibility, resulting in less reactive oxygen and weaker activity. Redox behavior from H_2_-TPR confirmed that CoCe­(WI), which shows low-temperature reduction peaks typical of highly reactive cobalt species, enabling efficient oxygen transfer and higher activity, whereas CoSi­(WI) and CoSi­(CP) exhibited weaker, broader peaks reflecting limited reducibility, which causes the low activity. The quantitative evidence of oxygen-vacancy concentration was derived from the surface O 1s XPS spectra and H_2_-TPR measurements. The O_ad_/O_latt_ ratio, representing the proportion of surface-adsorbed to lattice oxygen species, was 1.14 for CoCe­(WI) and 0.84 for CoSi­(WI) (Table 2). This higher ratio indicates a greater number of oxygen vacancies and mobile oxygen species on CeO_2_-supported catalysts. Similarly, H_2_ consumption of all prepared catalysts from Table 3 confirmed that CoCe­(WI) consumed 0.8693 mL g^–1^ STP, almost twice that of CoSi­(WI) (0.3908 mL g^–1^ STP), confirming its enhanced reducibility and capacity to supply reactive oxygen. These values are directly correlated with the CH_4_ conversion results as shown in (Figure 8), which confirms that catalysts with higher O_ad_/O_latt_ and H_2_ uptake exhibit superior CH_4_ oxidation activity. Apart from that, O_2_-TPD further confirmed that CoCe­(WI) released oxygen over a wider range, providing the mobile oxygen required for sustained CH_4_ oxidation, while SiO_2_-supported catalysts showed only weak desorption, consistent with their inferior performance. On the other hand, the O 1s spectra further confirmed that CoCe­(WI) contained lattice oxygen associated with vacancies that improve the CH_4_ activation, while CoSi­(WI) relied mainly on weakly bound adsorbed oxygen, consistent with its lower conversion. These results highlight that both the synthesis method and the choice of support play critical roles in modification for the structural and surface characteristics of Co-based catalysts, which ultimately determine their performance in CH_4_ oxidation. Overall, the results from XRD, Raman, XPS, H_2_-TPR, and O_2_-TPD together show a clear link between the structure and activity of the catalysts. These analyses consistently confirm that CoCe­(WI) has higher oxygen mobility, stronger metal–support interaction, and more surface-active oxygen species than the other catalysts. The agreement among all techniques supports the observed catalytic trends and validates the conclusions of this study.

## Conclusion

This study systematically assessed the influence of synthesis route and support nature on the physicochemical properties and catalytic performance of monometallic Co-based catalysts for low-temperature CH_4_ oxidation. The results demonstrated that the choice of support played an important role in tuning the dispersion of Co species, redox properties, and oxygen mobility. The CeO_2_-supported catalysts preserved the fluorite structure after Co incorporation, with Raman and XPS confirming lattice distortions and oxygen vacancies that promoted Co–support interaction and oxygen vacancies. As a result, CoCe­(WI) exhibited 91% CH_4_ conversion at 600 °C, while CoCe­(CP) achieved only 62%. In contrast, SiO_2_-supported catalysts exhibited weaker metal–support interactions and poor redox capacity, leading to moderate activities of 68% for CoSi­(WI) and 67% for CoSi­(CP). Further, the synthesis method also strongly influenced the structural and surface chemistry of the catalysts. The wet impregnation method produced catalysts with better Co dispersion, stronger metal–support interaction, and higher reducibility than coprecipitation, as confirmed by XRD, XPS, and TPR analyses. The stability test was performed for the best-performing catalyst, which showed minimal variation in conversion up to 12 h long run test under reaction conditions. The robustness of the best-performing catalyst is also examined through varying space velocity, which highlights the importance of operating conditions, with an optimum WHSV of 12,600 cm^3^ g^–1^ h^–1^ giving the highest conversion at 450 °C. These findings provide valuable guidelines for the design of cost-effective, non-noble metal catalysts with enhanced low-temperature activity for CH_4_ abatement and related environmental applications.

## Supplementary Material



## Notes

Figure S1: effect of different WHSVs on catalytic activity for best-performing catalyst CoCe­(WI) at fixed temperature 450 °C; Figure S2; point analysis of CoCe­(WI) and CoSi­(WI); Figure S3: XPS peaks for Ce 3d; Figure S4: XPS peaks for Si 2p; Figure S5: H_2_ TPR profile of bare support oxide in a flow of 5 % H_2_ balanced in He; Figure S6: O_2_ TPD profile of bare support oxide; Table S1: element composition of CoCe­(WI); and Table S2: element composition of CoSi­(WI) (PDF)

## §

Abu Dhabi Polytechnic University, Department of Petroleum Engineering Technology, Abu Dhabi, 111499, United Arab Emirates
