# Supplementary material for: Effect of Synthesis Routes and Support Nature on Co-Based Catalysts for Low-Temperature Catalytic Combustion of Methane
Source: ACS Omega. 2026 Apr 6;11(15):22606–20. doi: 10.1021/acsomega.5c10242 (PMC13103843; doi:10.1021/acsomega.5c10242)
Supplement: Supplementary file 1 [file ao5c10242_si_001.pdf]

Effect of synthesis routes and support nature on Co-based catalysts for low-temperature catalytic combustion of methane

Mirza Belal Beg,[1,] Labeeb Ali,[1,$] Suryamol Nambyaruveettil,[1] Abbas Khaleel [2]
Mohammednoor Altarawneh[1*]

[1]United Arab Emirates University, Department of Chemical and Petroleum Engineering,
Sheikh Khalifa bin Zayed Street, Al-Ain 15551, United Arab Emirates
[2]United Arab Emirates University, Department of Chemistry,
Sheikh Khalifa bin Zayed Street, Al-Ain 15551, United Arab Emirates

Corresponding author Email:

*mn.altarawneh@uaeu.ac.ae

$ Present address: Abu Dhabi Polytechnic University, Department of Petroleum Engineering Technology, Abu Dhabi, 111499, United Arab Emirates

<u>**Supporting Documents**</u>

Effect of WHSV on Catalytic Performance

To further evaluate the catalytic efficiency under different operating conditions, the influence of WHSV was examined for the best-performing catalyst, CoCe(WI), at 450 °C. As shown in Figure S1, the CH_4 conversion reaches a maximum at an intermediate WHSV of 12,600 $cm^3g^{-1}h^{-1}$, indicating the presence of an optimal operating condition. At higher WHSV values (16,200 and 23,400 $cm^3g^{-1}h^{-1}$), the conversion decreases, which is mainly attributed to the reduced residence time of the reactants in the catalyst bed, limiting the extent of CH_4 oxidation. This behaviour reflects an operational effect rather than an intrinsic enhancement of reaction kinetics, and the optimum WHSV represents a balance between reactant residence time and catalyst utilization.

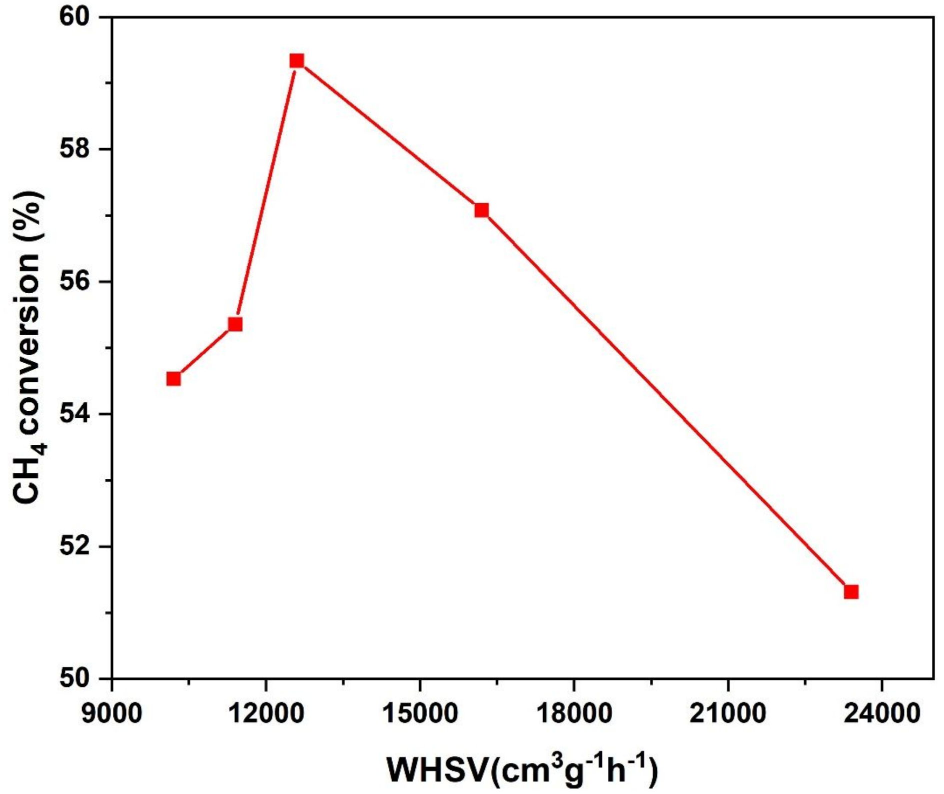

Figure S1- Effect of different WHSVs on catalytic activity for best-performing catalyst CoCe (WI) at fixed temperature 450°C.

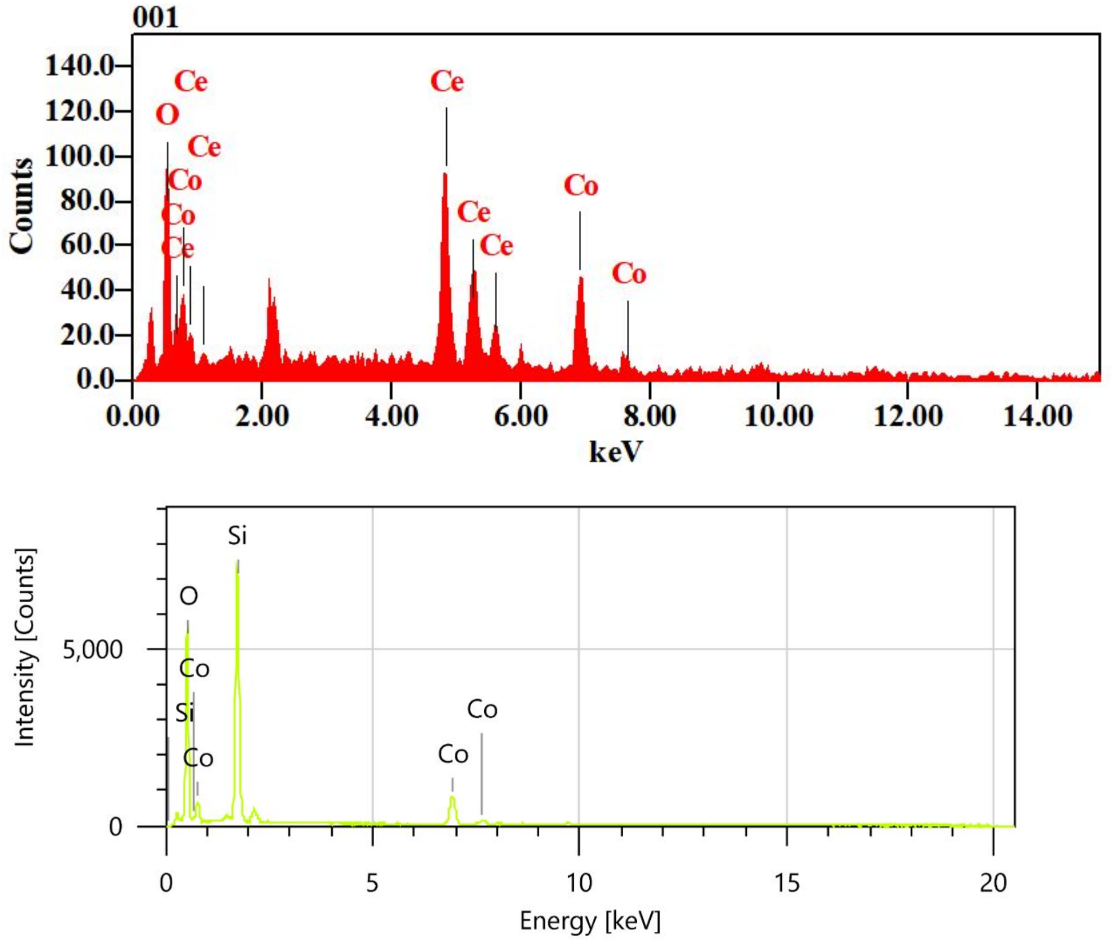

Figure S2- Point analysis of CoCe (WI) and CoSi(WI).

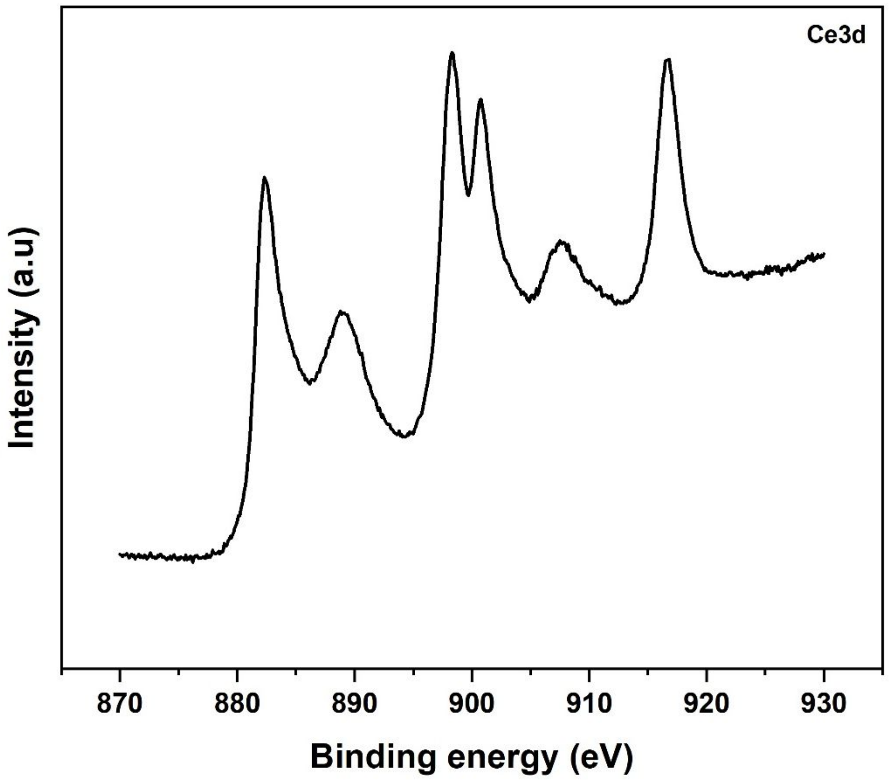

Figure S3- XPS peaks for Ce3d

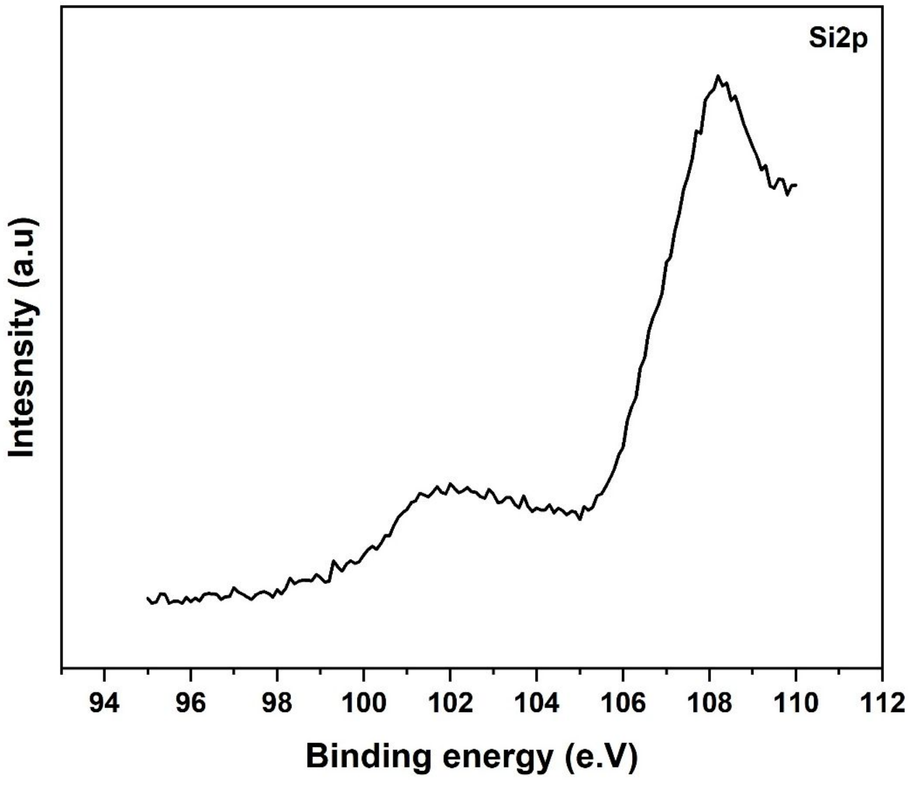

Figure S4- XPS peaks for Si2p

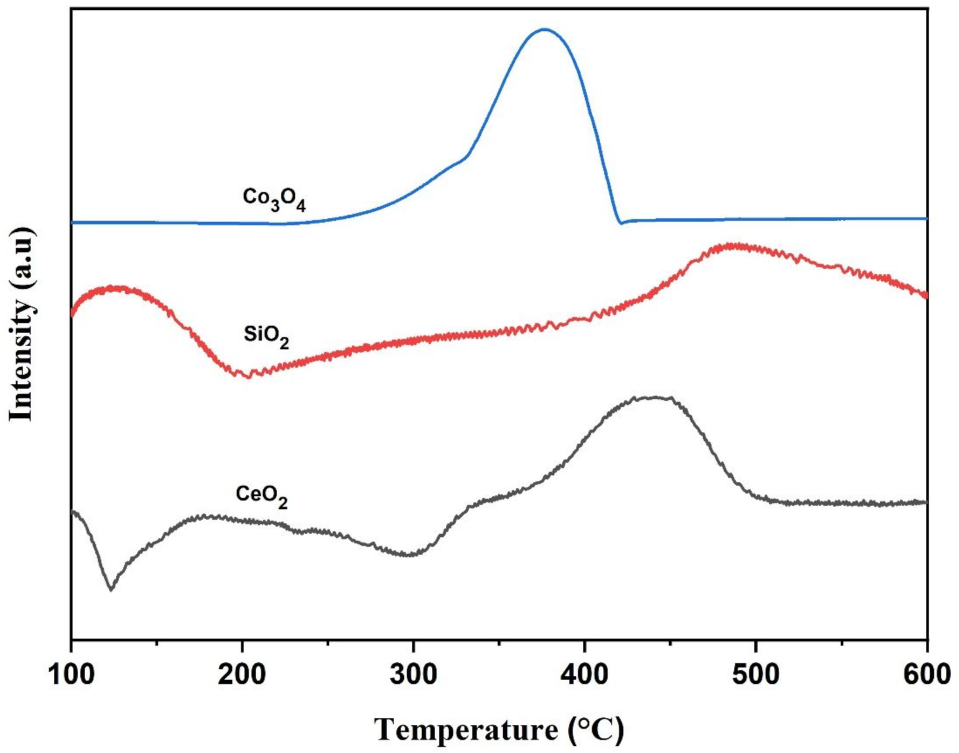

Figure S5- H_2 TPR profile of bare support oxide in a flow of 5 % H_2 balanced in He.

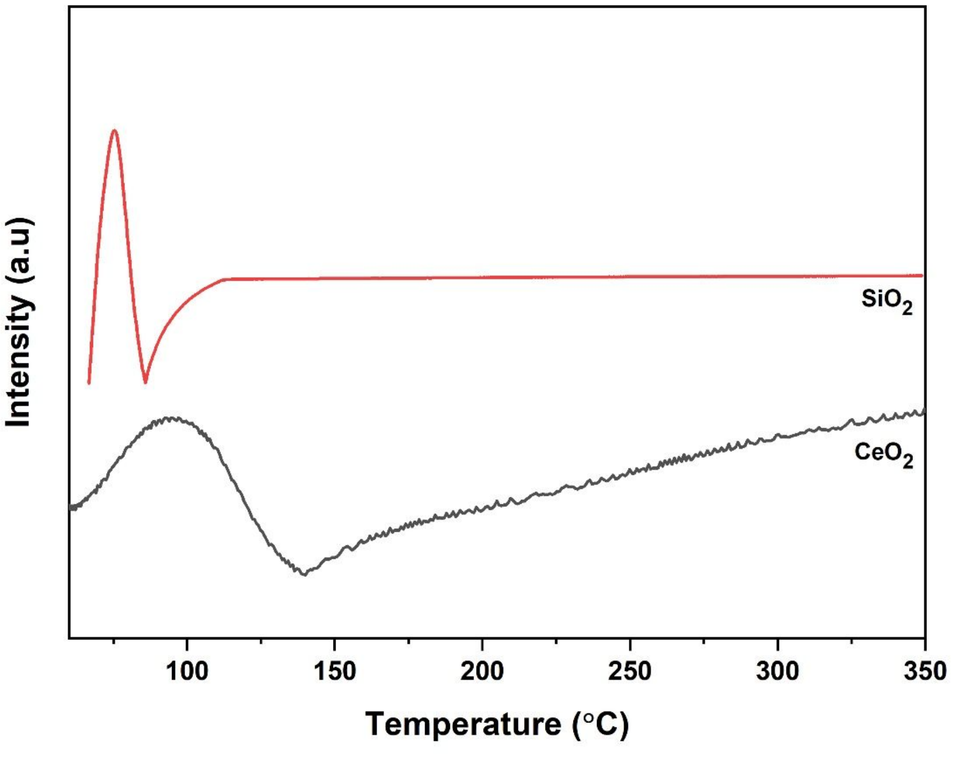

Figure S6- O_2 TPD profile of bare support oxide.

Table S1- Element composition of CoCe (WI)

Formula	mass%	Atom%	Line
O	15.70	54.01	K
Co	23.81	22.23	K
Ce	60.49	23.76	L
**Total**	100.00	100.00	

Table S2- Element composition of CoSi (WI)

Formula	mass%	Atom%	Line
O	49.14	67.83	K
Co	19.83	7.12	K
Si	31.86	25.05	K
**Total**	100.00	100.00	
